# Medication adherence in allergic diseases and asthma: a literature review

**DOI:** 10.3389/fphar.2024.1488665

**Published:** 2024-12-02

**Authors:** Elizabeth Malaya, Adrianna Piątkowska, Michał Panek, Piotr Kuna, Maciej Kupczyk, Grzegorz Kardas

**Affiliations:** Department of Internal Medicine, Asthma and Allergy, Medical University of Lodz, Lodz, Poland

**Keywords:** medication adherence, asthma, allergic diseases, interventions, assessment

## Abstract

Patients’ collaboration with healthcare providers, along with their individual dedication to follow medical recommendations, is a crucial component of effective therapy in chronic diseases. If a patient fails to fill their prescription, administers the medication improperly in terms of method and/or dosage, misses follow-up visits, or discontinues the treatment for any reason, these lapses can adversely affect disease management, impairing the effectiveness of symptom relief and prevention of progression and complications. A comparable situation pertains to allergic diseases, which require long-term and consistent treatment to achieve symptom alleviation and control. Research has shown that adherence rates for long-term therapy in chronic diseases have improved marginally over the years and continue to hover at approximately the figure published in a World Health Organization (WHO) report “Adherence to long-term therapies: evidence for action.” from 2003, which had stated that only 50% of patients in developed countries follow medical recommendations and that this rate would be even lower in developing countries. Over 20 years later, literature indicated that there has been only a slight improvement on the matter, leaving room for developing and implementing effective solutions to improve medication compliance. Further investigation on this matter is required. Causes for non-adherence classified by the Global Initiative for Asthma in their main report seem to correspond to those of the report by the WHO. Similar dependency might be determined by other allergic diseases as they fit chronic disease criteria, and the issue of non-adherence affects them too. This literature review seeks to compile and synthesize current insights on factors that influence adherence, as well as explore potential methods for monitoring, evaluating, and improving its outcomes in chronic diseases related to the medical field of allergology, such as asthma, allergic rhinitis, allergic conjunctivitis, rhinoconjunctivitis, atopic dermatitis, and urticaria.

## 1 Introduction

The therapeutic success in chronic diseases, even with an accurate diagnosis and the implementation of current guidelines, cannot be fully achieved without patient cooperation and willingness. The degree to which a patient follows medical instructions plays a crucial role in predicting the effectiveness of symptom management, preventing disease progression, and improving the overall quality of life (QoL). This statement does not introduce a novel finding, as Hippocrates at approximately 400 BC already noted that some patients had complained about unsuccessful treatment after inconsequences with prescribed medication (Vrijens et al., 2012).

The World Health Organization (WHO) in 2003 defined the term “adherence” as “patients following medical instructions.” This definition includes not only the patient’s initiative to follow recommendations solely but also whether they are able to use medications in a proper way (method and/or dosage) (World Health Organization, 2003). It is also possible to use the term “compliance” interchangeably, as their definitions are similar. “Poor adherence,” on the other hand, is defined as the failure of treatment to be taken as agreed upon by the patient and the healthcare provider (HCP) (Global Initiative for Asthma, 2024). With growing interest in the subject, “adherence” has been further redefined by extending the concept with “primary” and “secondary” distinguishers. Therefore, “Primary adherence” refers to the initiation of a new medication, such as filling prescriptions, while “secondary adherence” refers to the continuation of the treatment, for example, actions decreasing it could be self-limiting the treatment or using a different dosage than recommended of a drug already acquired (World Health Organization, 2003; Lam and Fresco, 2015). The “primary medication non-adherence” (PMN), another term in the literature, refers to a scenario in which the first prescription for a new medication was not fulfilled for a specific period since the day of its issuance (Schnorrerova et al., 2024).

In 2003, the WHO reported an adherence assessment conducted among patients suffering from chronic diseases and found it as being only 50% in developed countries, estimating it to be even lower in developing ones (World Health Organization, 2003). Recent adherence to medication has shown slight improvement over the years, yet it was still concluded to remain low, with 55.5% in Spain (Fernandez-Lazaro et al., 2019), 43.1% in Saudi Arabia (AlHewiti, 2014), 31.9% in Ethiopia (Ketsela Zeleke et al., 2024), 59.4% in North West Ethiopia (Kasahun et al., 2022), 51% in India (Tolley et al., 2023), and 50.31% in Iran (Dabaghian et al., 2016). Over the years, research has made some progress in this area, but it has rendered the pertaining issue of non-adherence obstructing successful treatment and its demanding need for effective intervention. The possibility of similar dependency has come to our attention, as allergies are among the most common chronic conditions with an immense variety of symptoms, ranging from mild and neglected to severe with high risk of exacerbations.

Allergic diseases are defined as a hypersensitivity reaction initiated by proven or strongly suspected immunological mechanisms, which could be IgE-mediated or non-IgE-mediated. Reactions happen to individuals who are sensitized to allergens or a group of allergens (Tanno et al., 2016); for some patients, these reactions are hard to avoid, e.g., respiratory allergies. These conditions are often described as heterogeneous and sometimes might occur with IgE-mediated mechanisms and/or with type 2 inflammation. Asthma is a heterogeneous chronic respiratory disease, the allergic phenotype of which fits the described criteria, and as well as for other allergic diseases, its therapy relies on regular medication to control disease and prevent exacerbations. It is an important and widely prevailing condition, with its eosinophilic and th2-related types being frequent causes of severe disease progression and life-threatening exacerbations (Global Initiative for Asthma, 2024); thus, it was included in this review.

**FIGURE 1:**
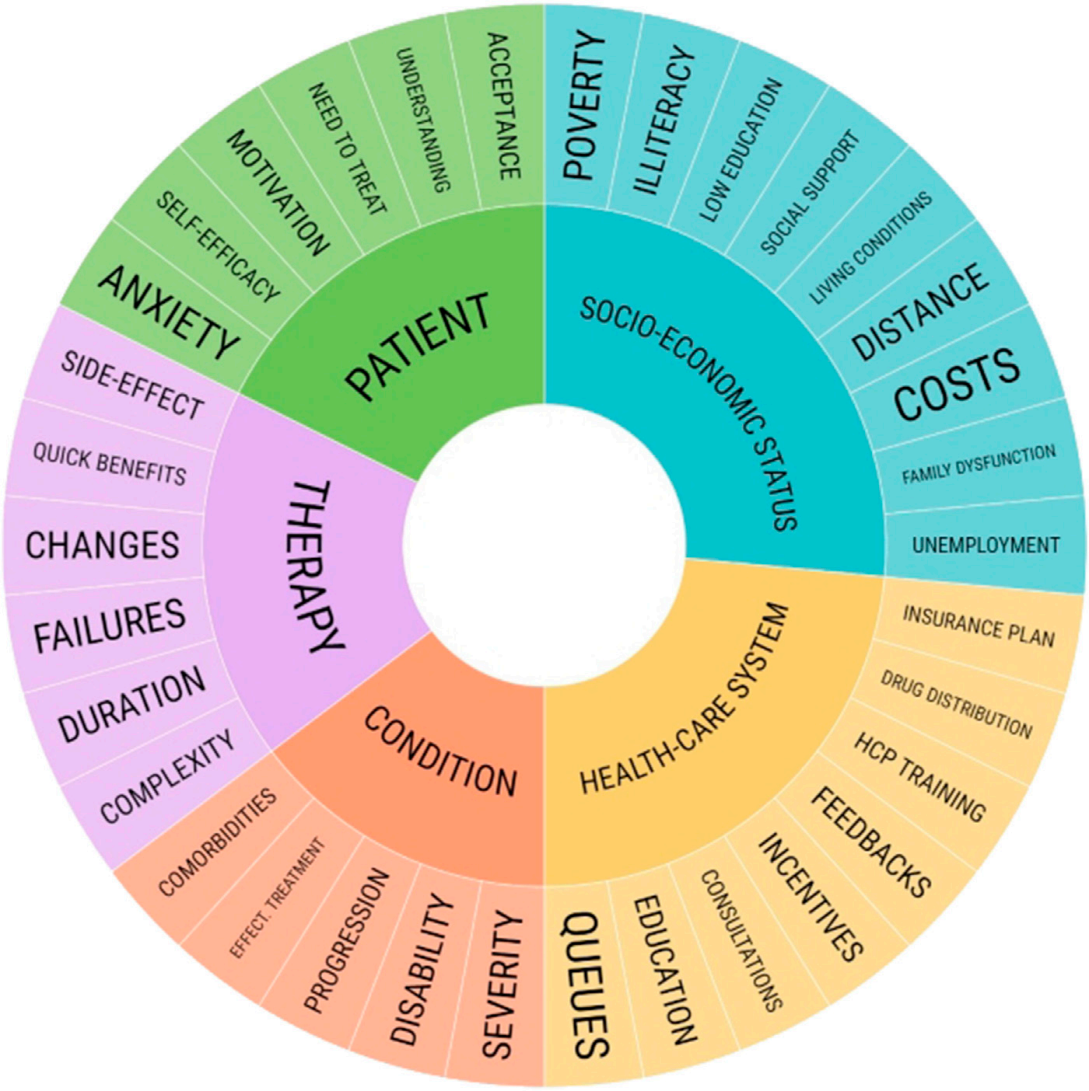
5 Dimensions affecting adherence.

There are many causes pertaining to poor adherence, which occur at every step of the treatment plan, from receiving recommendations and acquiring prescribed medication to successfully continuing the treatment until the next follow-up (Figure 1). These reasons for poor adherence had been grouped and compared to WHO’s “5 dimensions of adherence” classification, which include factors such as 1) socioeconomic status (e.g., living costs and conditions, illiteracy, level of education, employment status, distance to commune, poverty, and lack of social support); 2) therapy (e.g., complicated and demanding dosage regimen, frequent modifications of the administered treatment, previous treatment failures, necessity of regular and consistent drug administration for a long time, immediacy of beneficial effects or experienced adverse effects, and availability of medical support to deal with them); 3) patients (e.g., anxiety, self-efficacy, lack of motivation and self-perceived need for treatment, and misunderstanding and non-acceptance of the disease); 4) condition (e.g., severity and progression of disease, level of disability, availability of effective treatment, and comorbidities); and 5) healthcare system (e.g., HCP training, feedback and incentives, insufficient consultation, education, and long queues) (World Health Organization, 2003; Lam and Fresco, 2015). Factors determining poor adherence in chronic diseases are also encompassing those enlisted by the Global Initiative for Asthma (GINA) in its report for asthma. This classification of factors includes three groups: 1) medication/regimen-related factors, 2) unintentional, and 3) intentional poor adherence (elaborated further in the Asthma subsection). The strong connection between these two reports regarding the subject, as well as between the factors prevailing in recent literature regarding adherence in allergology has been highlighted; Table 1 lists possible reasons for non-adherence revised and summarized.

**TABLE 1:** Reported reasons for non-adherence in allergic conditions.

Factor group	WHO dimension and factor (Figure 1.)	Example	Intervention for improvement	Condition [source]
Medication regimen	Therapy: complexity	Frequency of administration	- Nudge-based interventions (non-monetary rewards and achievement unlocking) - Raising awareness of pros and cons - Self-monitoring and self-efficacy features - Extended-release drugs	Asthma Trojanowska et al. (2022); AR Park et al. (2023a); Park et al. (2023b)
		Amount of medication	- Combining drugs in one product (e.g., single inhaler and combined skin products)	Asthma Eikholt et al. (2023)
	HCS: drug distribution	Medication unavailable for outpatient care and frequent injections	- Facilitating home visits - Skill training of drug self-administration (e.g., subcutaneous shots)	AR Park et al. (2023a); Park et al. (2023b)
	Therapy: duration	Prolonging therapy with unsatisfying effects	- Nudge-based interventions (e.g., motivating and informative mHealth features)	AC and ARC Di Bona et al. (2020)
	Therapy: side-effect or previous failures	Bad experience and anticipatory anxiety	- Open discussion about patient’s concerns - Facilitating bias-free information flow	AR Wise et al. (2023)
Unintentional	Patient-related: anxiety	Corticophobia and other concerns		AD Gomes et al. (2022); Zorko et al. (2023); Albogami et al. (2023)
	HCS: HCP training and education	Insufficient tertiary care in hospitals: understaffed institutions and unempathetic staff	- Training of HCPs - Coordinated care	AR BG et al. (2024); AD Davis et al. (2021)
	HCS: consultations	Excluding patient’s preferences	- Including patient in decision-making	AD Ragamin et al. (2023); van der Kraaij et al. (2020); AC, AR Manzotti et al. (2016)
	Socioeconomic: illiteracy	Misunderstanding: drug misuse (technique or dosage) and misinterpretation (guidelines or recommendations)	- Demonstrations and skill training - Self-monitoring - Objective monitoring - Simplified instructions - Nudge-based interventions (e.g., motivating and informative mHealth features)	Asthma Eikholt et al. (2023); AD Ragamin et al. (2023)
	Condition-related: comorbidities	Chronic diseases, nicotinism, HIV/AIDS, drug and alcohol abuse, and psychiatric disorders	- Treating comorbidities - Coordinated care	Asthma Xia et al. (2023)
	Socioeconomic: distance	Long distance, commuting, and queues	- Facilitating home visits - Telemedicine	AR Park et al. (2023a); Park et al. (2023b)
	Socio-economic status: poverty and costs	Avoiding purchase and/or skipping doses to save money	- Cheaper generic drugs - National reimbursement systems	Asthma Xia et al. (2023); Margolis et al. (2022)
Intentional	Therapy: quick benefits, need to treat, and acceptance of the disease	Stopping medication with symptom alleviation	- Raising awareness to exacerbation risks - Explaining goals of treatment - Nudge-based interventions	Astma Cvietusa et al., (2023) Urticaria Kaneko et al. (2015)
	Patient: motivation; HCS: incentives Condition: low effectiveness of the treatment	Ceasing medication due to unsatisfactory results		Asthma McQuaid et al. (2003); AR Park et al. (2023a); Park et al. (2023b)
	Patient-related: acceptance and understanding	Self-provided treatment against recommendation	- Regular follow-ups (monitoring and feedback by HCP) - Explaining the risks of self-medication	AR Bousquet et al. (2023)
	Patient: self-efficacy	Forgetfulness	- Nudge-based interventions	Asthma Strandbygaard et al. (2010); AR Fox et al. (2023)

Apart from knowing the factors inflicting adherence, it is also important to seek reliable data on its assessment, which helps plan not only proper treatment regimens but also adequate communication and education strategy for HCPs in general practice (World Health Organization, 2003). The WHO identifies three methods for measuring adherence: 1) subjective ratings by providers and patients, 2) standardized, patient-administered questionnaires, and 3) biochemical measurement (World Health Organization, 2003). These methods range from subjective to objective, as they can depend on a patient’s individual impression or data gathered independently like, for instance, e-prescription data. They can also be differentiated into direct methods (e.g., drug or biomarker blood concentration) and indirect methods (e.g., pill counts, database research, and self-reports) (Kreys, 2016; Lehmann et al., 2014). Some methods applied to assess adherence in allergology were highlighted and are collectively summarized in Table 2. Since the WHO report of 2003, there is still no well-established “gold standard” for adherence assessment (World Health Organization, 2003), although many methods have been tested throughout the years. One of the most common and cost-effective methods for adherence assessment is self-reporting. The Morisky Medication Adherence Scale (MMAS-8 or MMAS-4) is often the questionnaire of choice, being established as a “gold standard,” and is readily available at healthcare centers (Moon et al., 2017; National Collaborating Centre for Primary Care UK, 2009).

**TABLE 2:** Adherence assessment tools reported in allergic conditions.

Method	Example	Pros	Cons
Questionnaires	- MMAS-4 and -8	- User-friendly	- Subjective
	- MARS	- Cost-effective	- Tentative evaluation
mHealth	- Apps: MASK-air Allergy Monitor	- User-friendly	- Possession and certain skills using a smartphone device are required
	- Websites: e-diaries, self-monitoring portals	- Easy real-time data collection and storage	- Not commonly in practice
	- Smart-watches	- Secure information	- Availability
		- Entertaining and informative	- Cost
		- Nudge-based interventions applicable	
		- Mostly cost-effective	
eHealth	- Smart inhalers	- Feedback	- Cost
		- Real-time adherence monitoring	- Availability
	- e-prescription data	- Primary adherence monitoring	- Cannot confirm drug administration even if purchased
Direct assessing (lab tests and physical test)	- Blood test (e.g., serum drug levels)	- Applicability for previous non-adherence (FeNO)	- Result bias due to other conditions
	- Other parameters	- Monitoring adherence to OCS	- Not for outpatient care

To measure adherence, the World Pharmacy Council had advocated the proportion of days covered (PDC) method, which is the number of days when a drug was available to the patient divided by the number of days in the study period. It can be calculated by using pharmacy dispensing records. Another method is the medication possession ratio (MPR), which is the percentage of day’s supply obtained during a specific time. The PDC more accurately reflects patient adherence, with handling drug switching and prescription overlaps more effectively. It is also a preferred method to measure adherence for chronic therapies (World Pharmacy Council, 2020).

We have examined and presented recent conclusions in scientific literature, regarding the problem of non-adherence to medication for common diseases strongly familiar to the field of allergology, such as asthma, allergic rhinitis, allergic conjunctivitis, rhinoconjunctivitis, atopic dermatitis, and urticaria.

## 2 Asthma

Asthma is usually characterized by chronic airway inflammation, with a history of respiratory symptoms, such as wheezing, shortness of breath, chest tightness, and coughing. Symptoms vary in intensity and over time are accompanied by expiratory airflow limitation. As there is no causal cure for asthma, its treatment is multimodal and mainly focuses on proper symptom management according to the (S)MART plan, requiring controller drugs (long-acting bronchodilator (LABA) and/or inhaled corticosteroids (ICS) applied daily even without concurrent symptoms) and anti-inflammatory relief drugs (short-acting bronchodilator (SABA) and ICS, which can be administered as needed). These elements can be administered in a single inhaler to facilitate regular administration, which stands for “S” in the name of the treatment plan (Global Initiative for Asthma, 2024).

Personalized asthma management, according to GINA, consists of three continual steps, which are repeated with each follow-up: 1) assess (confirmation of diagnosis if necessary, symptom control and modifiable risk factors, comorbidities, inhaler technique and adherence, patient preferences, and goals); 2) adjust (treating of modifiable risk factors and comorbidities, pharmacological and non-pharmacological strategies for treatment modification, education, and skill training); 3) review (reassessment of treatment outcomes like symptoms, exacerbations, side-effects, lung function, comorbidities, and patient satisfaction). Although “adherence” is mentioned once, all three stages are associated with patient collaboration and may inflict overall adherence (Global Initiative for Asthma, 2024). Based on the effectiveness of the treatment administered, various clinical subtypes are distinguished, such as 1) uncontrolled asthma, 2) difficult-to-treat asthma, and 3) severe asthma. Uncontrolled asthma is characterized by poor symptom control and/or frequent or severe exacerbations. Difficult-to-treat asthma is a subtype of uncontrolled asthma, with modifiable factors such as incorrect inhaler technique, poor adherence, smoking, comorbidities, or incorrect diagnosis. Severe asthma is a subset of difficult-to-treat asthma. It refers to asthma that remains uncontrolled despite adherence to maximal optimized high-dose ICS-LABA treatment and management of contributing factors, or that worsens when high-dose treatment is decreased (Global Initiative for Asthma, 2023). The treatment strategy for those subtypes differs significantly; for instance, difficult-to-treat asthma would require proper education, comorbidity treatment and management of modifiable risks in a first line of treatment, while assessment of the severe type would result in modifying the treatment aimed directly at asthma, using leukotriene receptor antagonists (LTRA) or biologic therapy. Therefore, proper assessment of adherence has a decisive impact for planning asthma management strategies (Global Initiative for Asthma, 2024). Poor adherence is one of the several independent exacerbation risk factors that have been identified and assessed in primary care. Other factors mentioned are history of exacerbation in the previous year, incorrect inhaler technique (part of the adherence term regarding misusing medication), chronic sinusitis, and smoking (Global Initiative for Asthma, 2024). To identify poor adherence in clinical practice, GINA recommends framing questions with empathy toward the patient to acknowledge the likelihood of incomplete adherence and to foster open, non-judgmental discussion. Monitoring medication through dosage counters on inhalers or electronic drug dispensing records can also be helpful (Global Initiative for Asthma, 2024). MMAS-8 or MMAS-4 is also widely utilized. GINA also lists a few methods for objective assessment, such as monitoring of prescribing or dispensing records, blood prednisone levels, and electronic inhaler monitoring (Global Initiative for Asthma, 2024).

There are different types of digital technologies classified as electronic, including mobile healthcare services (eHealth and mHealth), such as smartphone or tablet applications (apps), digitized respiratory inhalers, mobile spirometers, and other digital devices used to measure respiratory physiologic (to compensate for the absence of physical examination and in-laboratory pulmonary function testing) or health parameters (oximeters and activity monitors) (Kouri and Gupta, 2023). Standalone apps available on smart devices are increasing in number and variety in services provided; however, despite an aesthetically pleasing interface, many of them are designed without the involvement of practicing healthcare experts and lack features implementing official guidelines. The lack of engaging and informative elements degrades them in the Mobile App Rating Scale (MARS) (not to be confused with Medication Adherence Report Scale); another issue reported was limited customization, causing them to be non-personalized for individual needs (Robinson et al., 2024). An overwhelming number of available software does not represent practical benefits. Among 422 apps evaluated in the studies and available on the Apple App Store and Google Play Store, not many over 53 were assessed eligible, being related primarily to asthma and enabling bug-free functionality, with use of English language. However, implementing few features does not guarantee their efficacy; however, professional insight could improve their value. Apps that involved HCPs during the designing phase demonstrated a significant ability to facilitate behavioral change, such as education, self-monitoring or tracking features, self-efficacy, motivation, goal setting, information, or education techniques like tips, strategies, and skill training elements (Robinson et al., 2024). The applicability of the validated apps has been studied as well. The Mobile Airways Sentinel Network app (MASK-air/MASK-rhinitis - currently validated for allergic rhinitis) has been evaluated to observe whether it could be used for asthma. This application enables collecting daily visual analogue scale (VAS) data within e-diary entries for nasal and ocular allergy symptoms. Asthmatic symptoms such as shortness of breath had been included. The results had established no significant correlation between adherence to the app and adherence to the asthma medication, proving that this tool is not yet ready but providing the basis for further investigation in this direction, proving possible advantages (Roth et al., 2022). Among other evaluated examples is the ADolescent Adherence Patient Tool (ADAPT), which focuses on education, motivation, and self-monitoring elements. This app had a slight effect on children with initially poor adherence; however, despite higher compliance, their quality of life did not improve considerably. The result was not visible in children who had good adherence in general, as there was not much room for improvement to begin with. Intervention involving this app could make an impact on adolescents with poor adherence (Kosse et al., 2019). Proper design with verified knowledge assets could be gained from integrating with electronic medical tools (Robinson et al., 2024). Electronic monitoring devices (EMDs, also called Electronic Adherence Devices) for asthma adherence include next-generation digital inhalers that are integrated with mobile apps, that are capable of collecting objective data providing insights not only into whether a medication has been, indeed, taken by the patient but also the effectiveness of the administration technique (Eikholt et al., 2023; Exarchos et al., 2022). Nevertheless, EMDs may still be perceived as unavailable or too expensive for some patients (Eikholt et al., 2023; Toelle et al., 2020). Data indicate that interventions to improve adherence using EMD inhalers are cost-effective and provide good value for money, implying that some of these unnecessary expenses can be attributed to inhalation technique mistakes (Eikholt et al., 2023).

Another objective attempt to measure adherence to inhalable therapy in asthma studied in the field was the fractional concentration of exhaled nitric oxide (FeNO). This marker increases in response to airway inflammation associated with type 2 asthma and other conditions such as eosinophilic bronchitis, atopy, allergic rhinitis, and eczema. It is not validated as a diagnostic tool for asthma (Global Initiative for Asthma, 2024); however, it has been observed that the suppression of high FeNO levels after 5 days of directly observed therapy was an indicator of poor adherence in the past (Global Initiative for Asthma, 2024). The meta-analysis by Alahmadi et al. (2021) on the use of exhaled FeNO does not endorse its effectiveness as an objective and direct assessment tool for adherence. Although FeNO levels decrease with the use of ICS and are lower in patients who adhere to the treatment, no reliable cut-off exists that permits the accurate classification of present adherence. Further studies are required to explore the potential of using this marker to identify past non-adherence. As for other blood markers, the measurement of blood prednisolone levels with or without serum cortisol is commonly used as a direct, objective measurement method. Current results often suggest poor adherence to oral corticosteroids (OCS) even within clinical trials.

Adherence with asthma treatment is estimated to be rather suboptimal; approximately 50% of adults and children on long-term asthma therapy fail to take medications as directed at least part of the time (Global Initiative for Asthma, 2024). Adherence is extremely low with maintenance ICS: for adolescents and young adults, overall it was 28%. Those aged below 18 years had a slightly higher score of 36% (Global Initiative for Asthma, 2024). A certain role in this setback is played by the patient’s mindset toward ICS and corticosteroids (CS) in general, as more often the term “Corticophobia'' prevails in literature, especially among parents of children affected with the disease. It represents a patient’s fears and concerns about the adverse effects caused by corticosteroid medication, which are often propagated by media misinformation, as well as insufficient information about the pros and cons of such therapy provided by HCPs (Gidaris et al., 2021). Moreover, 44.44% of parents in Poland admitted to having concerns about medicating their children with ICS and OCS (Trojanowska et al., 2022). Adherence improves with symptom development (Cvietusa et al., 2023), including ICS medication. Based on electronic medical data of asthma patients of all ages, who had experienced exacerbation in the previous 12 months, primary adherence to controller medication was assessed as poor and reported to be better to that of ICS (86%) than to the LTRA’s (80%) or ICS/LABA (82%) combination, when prescribed for the first time. Adherence was worsening with ICS during long-term therapy. It was also noted that Latino and Black populations were less likely to fill prescriptions (Wu et al., 2015). The preference over other drugs compared to ICS has also been studied; for instance, ICS paired with LABA other than formoterol had better adherence; however, it could not lower the frequency of using the SABA reliever and neither had any impact on control over the condition (Sousa-Pinto et al., 2023). To monitor how many prescriptions fail to be filled, the literature often prefers to estimate non-adherence instead of adherence. Kardas et al. (2020) conducted a study in Poland analyzing e-prescription data, according to which more than 1 out of 7 prescriptions were not filled. PMN adherence significantly differed by age but not gender. The highest adherence rate was in the group aged 65–74 years and lowest among older patients, aged 75+. The highest primary non-adherence was observed for ICS + LABA combinations (18.86%), with an overall rate of 15.3% for inhaled medicines.

There is a wide range of factors that tend to inflict adherence in asthma. GINA specifies three groups of factors contributing to poor adherence: 1) medication/regimen (difficulties in use of inhaler, burdensome regimen, and multiple inhalers); 2) unintentional poor adherence (misunderstanding about instructions, forgetfulness, absence of daily routine, and cost); 3) intentional poor adherence (perception that treatment is not necessary, denial or anger, inappropriate expectations, concerns about side-effects, dissatisfaction, stigmatization, cultural or religious issues, and cost) (Global Initiative for Asthma, 2024).

Other studies support GINA’s rapport in terms of reasons for poor adherence; for instance, regarding medication and regimen causes, it has been noticed that approximately 44.43% of the asthmatic children are reluctant to take medications every day, but 16.67% have problems with using their inhalers (Trojanowska et al., 2022). Regarding unintentional factors, it is demonstrated that economic status seems to be one of the reasons for low adherence among asthma patients, as well as in other medical fields. According to the National Health Interview Survey (NHIS) in the USA, approximately 10% of patients with a history of asthma skipped doses to save money, 10% took smaller dosages, and over 13% delayed filling up a prescription (Xia et al., 2023). Moreover, studies among parents of children with asthma have revealed that lower asthma control rates are more likely to happen in households with a lower annual income (Margolis et al., 2022). In Kyrgyzstan, poor adherence to inhalers was a problem for 81% of patients, with 68.3% of them reporting that it was due to financial reasons, and 80% of the studied population from this region was assessed with poor adherence (Tabyshova et al., 2022). Poor drug distribution, its absence in pharmacies, and high costs significantly contribute to non-adherence in low- to middle-income countries (Desalu and Ozoh, 2021); for instance, the assessed accessibility in India was 30.1% and 43.1% in commercial and public sector medications for asthma management, respectively, which could lead to three times higher mortality rates in this country, as reported by Swarnakar and Dhar, (2024). According to the NHIS, adherence was worse for patients with lower education, who were uninsured, and had multiple comorbidities (like diabetes, hypercholesterolemia, or hypertension). Active smokers were less likely to adhere to treatment too (Xia et al., 2023). One more significant factor for adherence was age. In the case of pediatric patients, older children are more likely to have poorer adherence, despite their higher knowledge and understanding of disease. It is possible that one of the reasons is that parents tend to put more responsibility on them, but they lack motivation to keep up with treatment (McQuaid et al., 2003). Approximately 45.83% of the asthmatic children feel no need for medication when symptoms resolve or alleviate (Trojanowska et al., 2022). Pediatric patients have approximately 21%–68% lower risk of exacerbation, while the risk in adult patients decreases by 10% with every 25% increase in adherence (Engelkes et al., 2015). Conditions related to mental health correlate with chronic diseases and poor adherence, inflicting motivation to keep therapy in check. Asthma doubles the risk of being inflicted with depression in comparison with the rest of the population (Jiang et al., 2014). Asthmatic patients are also more likely to self-report depression and anxiety (Hurtado-Ruzza et al., 2021). Both depression and anxiety are associated with worse treatment outcomes, causing patients to suffer from difficult-to-treat asthma and be less adherent (Fong et al., 2022).

Efforts focused on studying and improving adherence continue to search for effective interventions. GINA cites several examples of successful interventions, including shared decision-making regarding medication choice, dosage, and frequency; proactive inhaler reminders or alerts for missed doses; reducing the frequency of drug administration (such as prescribing low-dose ICS once daily instead of twice daily); and increasing access to healthcare providers through home visits as part of a comprehensive asthma program (Global Initiative for Asthma, 2024). Daily messages with a prompt of medication doses can also improve adherence, as forgetfulness was cited as one of the most important reasons for its lower outcome. Patients that get the reminder take 18% more dosages, even though filling up the prescription is comparable in both groups (Strandbygaard et al., 2010).

## 3 Allergic rhinitis, conjunctivitis, and rhinoconjunctivitis

Allergic rhinitis (AR) clinically presents with nasal congestion, rhinorrhea, sneezing, postnasal drip, and/or nasal itching caused by airborne allergens, to which exposure can differ by time pattern, resulting in classification of AR as seasonal, perennial, or episodic (Bousquet et al., 2020; Wise et al., 2023). As allergic conjunctivitis (AC) and AR are frequently associated, their simultaneous manifestations generated a combined term rhinoconjunctivitis (ARC) (Iordache et al., 2022). There are well-established low-risk options to treat AR (over-the-counter (OTC) decongestants or cromolyns, prescribed ICS, antihistamines, and subcutaneous (SCIT) or sublingual immunotherapy (SLIT)). However, they require a patient’s engagement, not only in limiting exposure to allergens but also in drug self-administration. The appropriate selection of the treatment depends on the severity and frequency of the symptoms (Bousquet et al., 2020; Seidman et al., 2015).

Currently, known adherence assessment tools in AR include the standard Morisky scale and questionnaires available on mHealth apps such as MASK-air and Allergy Monitor. (Sousa-Pinto et al., 2022; Sousa-Pinto et al., 2024). MASK-air/MASK-rhinitis is validated for AR with the Medical Device Regulation (Class IIa) implementing patient-centered information and communication technologies (ICT) and is available and validated in approximately 22 countries (Bousquet et al., 2023; Menditto et al., 2019). Adherence to AR medication is variable (Fox et al., 2023); however, it is considered to remain suboptimal. The study with the use of MASK-air has reported an exceptionally low adherence. Approximately 70% of AR patients, who provided data through a quick questionnaire empowered by VAS scale for over 6 days, were non-adherent to medication. Only 11.3% were fully adherent (with MPR ≥70% and PDC ≤1.25). Based on other studies, it was also indicated that approximately 35% of patients were non-adherent for some time and 38% discontinued the treatment with symptom resolvement (Margolis et al., 2022). Data had also indicated that patients tend to alleviate symptoms with medication rather than adherence to a regular plan of treatment despite concurrent ailments. Self-applied treatment with oral antihistamine or other OTC drugs against provided recommendations was common (Bousquet et al., 2023). Keeping up with regular self-reporting methods was also found to be challenging owing to the long therapy duration. Patients with seasonal AR can maintain daily entries in their e-diaries for up to 2 months (Fraia et al., 2020). However, consistency with keeping records, as well as following healthcare instructions for a longer period, may vary.

One of the main reasons for non-adherence reported specifically for AR cases remains to be forgetting to use nasal sprays (daily intranasal corticosteroids and antihistamines) and/or nasal saline inhalation (NSI), which remain the basis for treating the symptoms of the disease (Fox et al., 2023). Therefore, memory triggers like nudge-based interventions (interventions aiming to change behavior in a predictable way like short messaging service (SMS) reminders, monetary and non-monetary rewards, mobile application-based interventions, and low-technology reminders to place in the home) were reported to be the most effective facilitators of adherence in this case. Counseling, a discussion of common side-effects and potential interventions during exacerbations and a demonstration on how to effectively use medication, might be helpful (Fox et al., 2023). Use of mHealth applications enabled with proper software might provide solutions to problems mentioned above by providing cost-effective and secure information flow and communication as nudge-based interventions are within app capabilities (Free et al., 2013). Mobile apps can enable cost-effective methods for secure information flow, communication with HCP, and nudge-based interventions (Free et al., 2013). Some studies have presented an interesting approach with the use of smart-watch as a promising tool for collecting real-time data on medication compliance in day-to-day life activity (Li et al., 2023). The other reason highlighted in recent studies was insufficient tertiary care in hospitals as patients receiving training on using nasal corticosteroids had better scores in their MMAS (BG et al., 2024).

AC primarily affects the conjunctiva. The classification of AC varies depending on the severity, frequency, and temporality, distinguishing simple allergy from perennial or seasonal subtypes and more severe vernal and atopic (Baab et al., 2024; Rathi and Murthy, 2017). Treatment consists of an eye-care routine involving antihistamines, vasoconstrictors, and mast-cell stabilizers administered with eye drops. For some refractory cases, eye drops may include CS for a maximum of 2 weeks under medical supervision. Oral antihistamines and corticosteroids are reserved for patients with systemic symptoms. Nonsteroidal anti-inflammatory drugs (NSAIDs) can also provide relief from symptoms (Baab et al., 2024). Untreated AC can impair vision, highlighting the importance of timely and effective intervention to reduce such risks (Labib and Chigbu, 2022). Mobile solutions had been tested for possible interventions, and the “Kayumidas–Itchy Eye Alert” app, providing push notifications of warning levels of itchiness based on function predicting pollen dispersal status, has been reported to effectively improve compliance with medication by applying changing behavior techniques, encouraging the use of eye drops, and increasing self-awareness (Mimura et al., 2023). This tool was another one of the nudge-based interventions with memory triggers presenting satisfactory results in improving adherence; however, the chronic course of the disease affects compliance with treatment, and proper education is required (Wadhwani et al., 2021).

In refractory cases, allergen-specific immunotherapy (AIT) is taken up for consideration (Seidman et al., 2015). AIT includes subcutaneous (SCIT) and sublingual (SLIT) therapies. Most studies revealed that real-world SCIT had persistence (continuation of therapy) and adherence rates of <80% (Park et al., 2023a), while real-world SLIT studies with longer follow-up had low rates, and 3-year adherence from 9.6% to 49.0% with 3-year persistence from 7% to 59.0% (Park et al., 2023b). Both SCIT and SLIT therapies allowed for a reduction in medicine use and AR symptoms. The effect of SLIT lasts for at least 2 years after a 3-year course of therapy. Harmful reactions to SLIT are minimal, with exceedingly rare systemic events, while reactions to SCIT consist of rare but severe systemic events, potentially fatal if ill-managed. The costs of SCIT and SLIT are moderate, and they are considered as cost-effective compared to pharmacotherapy (SLIT after years of administration). SLIT therapy seems to have lower total costs and is safer considering adverse effects (Wise et al., 2023). Among the reported reasons for SCIT therapy discontinuation were inconvenience (related to injection frequency, hospital visits, commuting, and waiting time), adverse reactions, poor efficacy, and cost. Reasons for SLIT discontinuation were similar, including inconvenience (reported forgetting doses and missing follow-ups), adverse effects, cost, and inefficacy. Symptom improvement was also common for SLIT discontinuation (Park et al., 2023a; Park et al., 2023b). The study conducted in Denmark highlights education level and age as reasons for noncompliance with AIT therapy. Within age groups, only SLIT therapy was higher for users aged 0–9 (Borg et al., 2021), which could be caused by the risk of adverse effects.

SCIT therapy for ARC is considered to be safe and well-tolerated, although less than half of the patients can complete 3 years of the treatment (Di Bona et al., 2020). A study in Denmark demonstrated a slightly better result in terms of adherence over this time period (SCIT 57% and SLIT 53%). However, compliance with both SLIT and SCIT was gradually decreasing each year (Borg et al., 2020). It is possible that involving the patient in the choice of the route of drug administration is associated with better compliance. Patients likely finish their 3-year course of treatment, when they are given a choice (Manzotti et al., 2016).

## 4 Atopic dermatitis

Atopic dermatitis (AD) is characterized by erythematous, itchy lesions of various morphologies, with periods of flare-ups and remissions. The location of those skin lesions in the body differs among age groups, but they are often found on flexural surfaces, such as elbows and knees. The disease often has an early onset affecting children (Frazier and Bhardwaj, 2020). Skin hygiene with the use of emollients (applied at least twice a day and after every shower) is considered the baseline therapy. Topical corticosteroids (TCS) are applied for flare-ups, and for more severe cases, they are combined with topical calcineurin inhibitors (TCI). For acute lesions “wet-wraps” with diluted TCS can be applied as a temporary treatment. Additionally, phototherapy is an option that can help with lesions (Frazier and Bhardwaj, 2020; Kulthanan et al., 2021).

Poor adherence to treatment can destroy the skin barrier, making it prone to infections and further leading to complications, such as systemic inflammation or sepsis (Wang et al., 2021). Adherence to a daily routine, requiring applying medications at least twice a day and after every shower, is very difficult to achieve (Kulthanan et al., 2021). Adolescents, in particular, spend either too little or too much time on their skin hygiene, which aggravates their condition and increases the risk of infection. Education was again listed as a crucial component of intervention (Wollenberg et al., 2020), and it is essential not only for daily routine but also to prevent the prevailing issue of corticophobia. Illiteracy fueled by misinformation spread in the media is significantly responsible for fear of CS (Gomes et al., 2022). The topical corticosteroid phobia (TOPICOP) questionnaire revealed that many patients are suffering from fear of TCS. Female patients exhibited higher levels of corticosteroid anxiety compared to men. The basis of this concern was unprofessional second opinion sought online (Zorko et al., 2023). Low efficacy and adverse effects contribute to the infamous opinion about CS. It has been noticed by 25.5% of parents of children suffering from AD that TCS had a brief period of efficacy after regular use. Blaming CS for causing the illness was among common beliefs to be reported (Albogami et al., 2023). The general concern was associated with the risk of causing skin discoloration and/or deformation; 44.5% of parents stopped CS treatment due to adverse events. The lack of awareness of pros and cons, as well as disagreement with the recommended treatment, indicate communication difficulties in primary care. It has been pointed out by the patients that the recommendations were unclear and not up to date. Adherence perceived by general practitioners in the Netherlands indicated the lowest results for the application of TCS and highest for emollients. Knowledge-related and patient’s attitude-related factors were strongly correlated to be at fault (Ragamin et al., 2023). Physicians lacking experience with CS in their practice have below-average confidence in prescribing them to patients (Andre et al., 2023), which also limits the rational medication strategy. For self-reporting methods, “Atopic App” was designed to evaluate the severity of illness using pictures sent by patients. It also contained Patient-Oriented Eczema Measure (POEM) questionnaires, scales for itch, tools to document triggers of AD, and web-based education program. The usage of the app was significantly higher among users who completed web-based courses (Zvulunov et al., 2023).

Several solutions for improvement have been considered. Many patients had expressed a desire for shared decision-making in their therapeutic process. However, despite dermatologists’ awareness of their preference, patients frequently felt unheard and excluded (van der Kraaij et al., 2020). This finding raises a potential need to encourage practitioners to involve patients in adjusting their treatment plan. It has been indicated that measuring transepidermal water loss (TEWL) using an evaporimeter and discussing the results with the patient would increase adherence to emollient therapy (Wollenberg et al., 2020). Including patients in therapeutic product-related decisions could help as well, but that does not guarantee a better adherence. The study compared two groups, each with a different product type applied (fatty ointment or cream formula), in terms of frequency and amount of medication administered. Overall, adherence was suboptimal, with approximately 48% of patients following the recommended regimen. Specifically, 42% of patients in the cream group adhered to the treatment, compared to 54% in the ointment group; however, the difference between groups was not statistically significant (Gregersen et al., 2024).

## 5 Urticaria

Urticaria is characterized by the development of wheals (hives) and/or angioedema (both present in 40%–50% of cases). Symptoms can last for more than 6 weeks or longer, based on which it is classified as acute spontaneous or chronic urticaria, respectively. Aside from triggers like plenty of allergies, urticaria can have hereditary or idiopathic origins. It may also be indicative of serious underlying conditions, such as autoimmune disease or cancer. Pregnancy or psychological distress could also be a trigger (Aslan Kayıran and Akdeniz, 2018; Zuberbier et al., 2018). Approximately two-thirds of cases resolve spontaneously, either at the beginning or within 24 h. H1-antihistamines administered once per day are within the first line of treatment. With refractory symptoms, dosage may be increased up to four times. Systemic corticosteroids (OCS) would be used next for up to 10 days, followed by cyclosporine (<2 days), and finally omalizumab, which is considered the last choice due to its excessive cost (Kayiran and Akdeniz, 2019).

A Singaporean study from 2015 investigated chronic urticaria (CU) and assessed whether the frequency of drug administration and, thus, adherence could improve QoL. The overall compliance rate was approximately 71.9%, with the majority of the tested population (25.2%) reporting medium adherence and 2.9% reporting high adherence. The frequency had no significant impact on the quality of life questionnaires (CU-Q2oL standardized for chronic urticaria). There was no substantial difference reported between patients with low and medium adherence either. Moreover, few patients with high adherence had their CU-Q2oL scores lower than the median (Heng et al., 2015). The study conducted in Japan around the same year used MMAS-8 to determine whether adherence for topical or oral medication differs. The group with topical medication had better mean scores on their MMAS; however, both groups were assessed with low adherence, with scores above 6 points; 11% of patients taking oral medication admitted to ignoring doctors’ recommendations, while 29% of patients taking topical medication discontinued their treatment when symptoms had alleviated (Kaneko et al., 2015). It has been found that patients with CU were mostly affected by disease, which interferes with their sleep and causes pruritus, resulting in daily fatigue, with female patients reporting the most discomfort. Women had been more affected by sleep interferences, embarrassment caused by conditions, and limitations on choice of the clothing material. The factors affecting patients the least were side effects from their medication and problems using cosmetics (Heng et al., 2015).

## 6 Summary

Asthma and allergic diseases are among the chronic diseases with their course depending on regular and rational medication, which may help prevent exacerbations and improve control over the condition. Symptoms can be aggravated when patients find treatment regimens incompatible with their lifestyles as some treatment plans may be demanding or difficult to understand. Whether the patient has followed the medical instructions or administers the medications properly may be assessed with measurement of adherence with the treatment plan provided by HCPs. Adherence is divided into primary and secondary adherence, as it appears to be significant in distinguishing whether the medication has been discontinued or has not acquired at all. There are direct (e.g., blood tests) and indirect methods (e.g., questionnaires), which can be implemented according to prevailing conditions, although it seems that there is still no well-established “gold standard” to accurately measure the adherence. All methods used in the reviewed literature are presented in Table 2.

Keeping up with the medication regimen is challenging with allergic diseases and asthma; skipping doses caused by own beliefs or by forgetfulness tends to be a prevailing issue. Asthmatic children are reluctant to take their medication every day, while AR patients often simply forget to use their nasal sprays or NSIs; in some cases, the doses have been skipped or reduced for economic reasons. Inconvenience (injection frequency, queues, and follow-ups) and costs are the most prevailing reasons for discontinuation of AIT to treat AR. Corticophobia is another issue inflicting adherence that has been observed among parents of asthmatic children and patients suffering from AD, as media misinformation tends to invoke the patient’s concerns and fears about adverse effects caused by corticosteroids. This form of aversion has been noticed among the patients with asthma and AD, but it can be present with other conditions including CS medication within the treatment plan. Adherence to the maintenance ICS in asthma is extremely low, but primary adherence to sole ICS seems to be better than to the other drug combinations; the worst adherence was noticed for the ICS + LABA combination. Regarding TCS, it has been noticed that female patients exhibited higher levels of corticosteroid anxiety than men. As for other factors responsible for poor adherence, the literature enlists comorbidities, age, socioeconomic status, race, or education level. The results of adherence inflicted by these factors vary; for instance, adherence to ICS is slightly worse among adolescent and adult asthma patient than those below the age of 18. Older children tend to have poor adherence despite their better knowledge of disease control. Primary adherence was significantly different between the age groups, while gender had no impact. Reasons for non-adherence reported in the reviewed literature are summarized in Table 1.

Successful methods to improve adherence consist of interventions including shared decision-making regarding the treatment strategy (medication choice, dosage, and frequency), reminder systems using EMDs and mHealth apps (SMS notifications and proactive reminders), reducing the frequency of drug administration, and increasing access to HCPs through home visits. Enhancing HCP education on effective communication with the patient could improve adherence and its assessment during each follow-up. mHealth tools such as standalone apps and digitized devices like respiratory inhalers and mobile spirometers are useful for measuring and improving adherence in pulmonary diseases. These tools can be used not only to collect data but also to improve drug administering techniques and patient motivation through the use of informative features and nudge-based interventions. The MASK-air app, which aims to improve adherence and symptom control in allergic rhinitis and could be used for asthma, is a good example of this. However, some of these devices are either not cost-effective or lack entertaining and bug-free functionality. Consultation with HCPs led by software developers is also one of the missing components in a significant number of available mHealth apps.

## 7 Discussion

For the purpose of this review, we have looked through the online National Library Center of Biotechnology Information (NCBI) database, with the greatest attention paid to the PubMed database and titles containing keywords such as “adherence,” “compliance,” “non-adherence,” “rhinitis,” “rhinoconjunctivitis,” “conjunctivitis,” “dermatitis,” “urticaria,” “Morisky,” and “mHealth.” Google Scholar, SpringerLink, ScienceDirect, LWW Health Library Medical Education, and Ovid had also been searched for acquiring more details on the reviewed literature. Included works had to be published in English, regarding human populations, and most recent with estimated 5–6 year tolerance (some works might be older due to no change in definitions, guidelines, or lack of elaboration on the matter such as urticaria adherence studies). All forms of research (reviews, meta-analysis, clinical trials, cross-sectional studies etc.), which provided insights on adherence matters regarding its assessment, inflicting factors, and interventions for improvement, were accepted. The exclusion criteria that meet the enlisted conditions and demonstrate that the publication is ineligible for review have not yet been determined. One term required further revision to decide whether it should have been used as a synonym with the term “adherence.” The term “compliance” originally appeared to correctly following therapeutic instructions without considering a patient’s willingness or motivation to cooperate. “Compliance” and “adherence” are not synonyms, but they are used interchangeably in the modern literature. “Adherence” is increasingly preferred to reflect a fundamental shift in the understanding of relationships between patients and HCPs. This term is thought to better evoke the idea of cooperation between the prescriber and the patient, rather than passive obedience (Vrijens et al., 2012). We have decided to use those two terms interchangeably as well, adapting to a more modern definition.

Medication adherence is recognized as one of the most impactful and cost-effective strategies for improving the health of the general population (World Health Organization, 2003). The issue of poor adherence still requires effective improvement approaches, and more reliable data could help visualize the problem and assess the effectiveness of interventions. The limitation was perceived, while reviewing assessment for medication adherence, as the proportional results, apart from GINA and WHO reports, had originated mostly from studies conducted on smaller populations, missing the global overall perspective. The potential bias could also be inflicted by the preference of no objective questionnaire method and lack of well-established “gold standard.” MMAS is still the most commonly used, cost-effective and efficient standardized questionnaire applicable for all chronic conditions, although subjective methods, such as those requiring some tolerance estimation, are likely to be utilized when analyzing results. The studies of non-adherence are not conducted repeatedly with the same environment and conditions on a regular basis. To compare the general medication adherence over time, results can only be compared to the observations with similar criteria, such as using MMAS assessment (graded low, medium, and high) on selected groups with all factors considered (without isolated factor or factors inflicting the score). Polls indicating factors, which are perceived the most as the potential cause by the patients or HCPs, can also be compared, but evaluation brings more tentative than accurate reliable proportions. EMDs as well as all electronic drug monitors, including medication event monitoring system (MEMS), allow tracing the number and frequency of the administered drugs. These mHealth tools are currently regarded as the “gold standard” for the purpose of objective adherence data collection, but mostly within a trial environment, due to the cost and lack of universality. Hardware and compatible software might be expensive, and devices are also limited to a single integrated product, with no monitoring for other generic drugs. They are also not established bias-free (Eikholt et al., 2023; Exarchos et al., 2022; Smith et al., 2010). Data provided by eHealth healthcare systems (HCSs) are limited to monitoring drug dispensations or registered follow-ups regarding monitoring adherence. E-prescription data should be the method of choice for the objective and effective monitoring of primary adherence to prescribed medication. Assessing secondary adherence heavily relies on the patient’s insight and willingness to share his observations, as well as on HCPs’ experience and alertness to identify non-adherence in practice.

Factors enlisted in the literature do not reveal novel observations in the matter of finding possible causes for non-adherence; WHO’s classification with its “5 dimensions” still encompasses the general areas of interest and can be used to classify factors in allergies alongside GINA’s classification for asthma, which points out mutual causes grouped in three categories. Both have been adapted to describe factors prevailing in allergic diseases and asthma, as shown in Table 1. Some of the factors would remain non-modifiable or could be altered to a little degree (therapy duration and complexity, with no alternatives available or comorbidities accurately diagnosed and treated, but refractory). Figure 1 presents factors prevailing in a reviewed literature, which are included on its outer circle, but is not fully exhaustive on the matter, and it is important to note that each dimension can have additional factors assigned or shared with other dimensions. For instance, disparities prevailing in societies around the world might inflict the socio-economic status, patient’s beliefs based on previous bad experiences, or healthcare systems based on HCP’s attitude or HCS policies. It has been reported that non-heterosexual and gender-fluid patients would often encounter discrimination from HCPs, which would discourage them to adhere (Udemgba et al., 2023). Inadequate reaction might not be due to HCPs’ lack of motivation and prejudice; HCPs from the United States had admitted to feeling uneducated enough to treat skin conditions affecting Afro-Americans (Davis et al., 2021). Homeless populations may suffer not only from limited access to HCPs and financial coverage issues but also from food scarcity, exacerbations, adverse effects from administered drugs, and less effective treatment, which all could lead to avoiding receiving help for the purpose of completing the treatment (Wilder et al., 2021).

Regardless of the method of grouping, it is apparent that the problem of non-adherence derives not only from the patient’s attitude or capabilities but also from the vast group of complex factors incapable to be resolved simply by endorsing education and discussion (socio-economic status-, healthcare system-, condition- and therapy-related factors). It is worth not neglecting the most significant importance of building professional trustworthy relations between the patient and his HCP, as well as endorsing literacy in both groups. Those aspects alone could cover most of the patient-related and healthcare system-related (e.g., in terms of proper HCP training) dimensions, and they should be considered the base of planning, initiating, and following the effective treatment plan. Enforcing HCPs’ with bedside manner skill training would support their work encounters with minority groups that could feel stigmatized or discriminated against. A more patient-friendly interpretation of the guidelines would be helpful in explaining the treatment, as traditional education provided by HCPs has proven to be too complex, requiring healthcare infrastructure and/or some extent of personalization to the patient’s individual needs in order to be cost-effective at a large scale (Nieuwlaat et al., 2014).

Smart technology applying mHealth employs the use of mobile phones, patient monitoring devices, personal digital assistants (PDAs), and other wireless devices enabling moderating utility of voice and text messages, notifications, quick and easy questionnaires, and information access (WHO Global Observatory for eHealth, 2011); nevertheless, the software catalog is overwhelmed with vast number of apps developed with insufficient informative qualities, lack of HCP insight, and features inefficient in behavioral change, making them inapplicable to healthcare recommendations and standardized guidelines (Robinson et al., 2024). Shifting the focus from numbers to quality to provide mHealth apps in cooperation with HCPs that implement actual guidelines in an easy and user-friendly manner could make a difference. Mobile apps can deliver information and enable education with motivation. They often use a broad range of methods enclosed in the term of behavior change techniques (BCTs), which aim to stimulate the user’s motivation and clarify all doubts about a patient’s condition and medication, including education, self-monitoring or tracking, advice or tips, skill training to improve drug administration techniques, action planning, perceived risks, and benefits (Robinson et al., 2024). Nudge-based interventions might achieve behavioral change too, which are defined as any intervention that would change behavior in a predictable way without limiting a patient’s individual freedom of choice (Fox et al., 2023). It can be achieved by easy and attractive health-promoting messages like SMS, letters, or brochures, which could contain reminders about check-ups, drug-administration, or self-monitoring. They can be used to promote healthy lifestyle habits and improve adherence (Murayama et al., 2023). A significant amount of research has been concluded with poor adherence assessment and apparent call for further investigation; however, few of the proposed interventions have been assessed and critically evaluated on more numerous, representative groups. Some of the proposed interventions remain to have little evaluation in real-life conditions, and their applicability relies on HCP’s decision based on the assessment of potential benefits and patient’s capabilities and preferences.

## 8 Conclusion

The review has been accomplished following the general conclusions: 1) although there is no definitive “gold standard” for objectively measuring adherence, the use of the MMAS questionnaire, enhanced by mHealth tools during follow-ups, can improve its evaluation and refine the medication strategy. Standardized questionnaires are considered cost-effective, straightforward, and user-friendly and should be the first line of adherence assessment, following an open, non-judgmental, and empathetic discussion with the patient. Effective communication with the patient is crucial for preemptive estimation. 2) Overall, adherence to medications in chronic allergic diseases remains low, exhibiting only gradual improvement over time. Better adherence rates are observed in developed countries that utilize eHealth technologies. In most cases, adherence decreases with the duration of the treatment. 3) Primary adherence tends to be higher than secondary adherence. The difference in this result may be explained by motivation derived from the patient’s expectation to alleviate currently occurring symptoms, as well as motivation boosted by the opportunity to test new medication. As the treatment continues, motivation gradually weakens because patients may not see satisfactory results, suffer from adverse effects, or have a complicated medication regimen that conflict with their lifestyle, and can discontinue the treatment when the symptoms alleviate. 4) Primary non-adherence, which accounts for the majority of the prescriptions that remain unfulfilled, can be easily and accurately determined by e-prescription data, provided by electronic systems integrated with healthcare medication dispensing. For the secondary adherence and adherence in general the Morisky Medication Adherence Scale remains to be the “gold standard” among standardized questionnaires; however despite being most cost-effective, their precision remains insufficient. 5) Although several interventions have been successful to improve adherence, there is still room for further innovation in this field. Key strategies to enhance patient adherence involve implementing reminder interventions and providing educational support. 6) Common reasons for poor adherence, frequently cited in the literature, include the discontinuation of treatment when symptoms subside, the fear of using medications containing corticosteroids, and poor medication administration techniques. These factors align with the causes identified in numerous studies over the years and can be mutual for the majority of chronic diseases.

## Glossary

AIT: allergen-specific immunotherapy
AC: allergic conjunctivitis
AR: allergic rhinitis
ARC: allergic rhinoconjunctivitis
AD: atopic dermatitis
BCT: behavior change technique
CS: corticosteroids
EAD: electronic adherence devices
eHealth: Electronic healthcare services
EMD: electronic monitoring devices
HCP: healthcare provider
ICS: inhaled corticosteroids
LABA: long-acting-beta2 agonists
MEMS: medication event monitoring system
MPR: medication possession ratio
MASK-air: Mobile Airways Sentinel Network for allergic diseases
mHealth: mobile healthcare services
MMAS: Morisky Medication Adherence Scales
NSI: nasal saline inhalation
NSAIDs: nonsteroidal anti-inflammatory drugs
OTC: over the counter
PDA: personal digital assistant
PMN: primary medication non-adherence
PDC: proportion of days covered
SMS: short messaging services
SCIT: subcutaneous immunotherapy
SLIT: sublingual immunotherapy
GINA: The Global Initiative for Asthma
TCI: topical calcineurin inhibitors
TCS: topical corticosteroids
VAS: visual analog scale
WHO: World Health Organization
